# Recent Advances in Vaginal Atresia: A Literature Review

**DOI:** 10.3390/biomedicines13010128

**Published:** 2025-01-08

**Authors:** Xijuan Lin, Jia Kang, Lan Zhu

**Affiliations:** Department of Obstetrics and Gynecology, National Clinical Research Center for Obstetric and Gynecologic Diseases, Peking Union Medical College Hospital, State Key Laboratory for Complex Severe and Rare Diseases, Peking Union Medical College, Chinese Academy of Medical Sciences, No. 1 Shuaifuyuan, Beijing 100005, China; xijuanlin@foxmail.com

**Keywords:** vaginal atresia, research advances, genetic factors, treatment strategies, fertility outcomes

## Abstract

Vaginal atresia is a rare anomaly of the female reproductive tract that significantly impacts women’s reproductive health and quality of life. Although there has been relatively extensive research on the clinical manifestations and differential diagnosis of vaginal atresia, there is a paucity of literature specifically addressing the genetic background, treatment protocols, and psychological status of patients with vaginal atresia, indicating a need for further investigation. In this context, this article systematically reviews the epidemiological characteristics of vaginal atresia and explores its etiology from multiple perspectives, including developmental processes, genetic factors, and environmental factors, emphasizing the importance of genetic susceptibility and environmental interactions in the pathogenesis of the condition. Building upon a summary of the clinical presentations, classification, and diagnostic methods of vaginal atresia, this article provides an overview of current treatment strategies for both partial vaginal atresia and complete vaginal atresia, discusses the psychological status of affected patients, and examines fertility outcomes and sexual function. The aim is to offer insights and recommendations for future research on vaginal atresia, ultimately striving to enhance the quality of life for affected individuals.

## 1. Background

Vaginal atresia is a rare anomaly of the female reproductive tract caused by abnormal development of the urogenital sinus and Müllerian ducts, leading to an incomplete vaginal canalization [1]; the incidence of vaginal atresia is estimated to be approximately 1 case per 10,000 to 15,000 female newborns [2]. Patients typically present with normal external genitalia, and a normal uterus, fallopian tubes, and ovaries, while experiencing partial or complete vaginal atresia. According to the updated classification system from the European Society of Human Reproduction and Embryology (ESHRE) and the European Society for Gynaecological Endoscopy (ESGE), for female reproductive tract anomalies, vaginal atresia is categorized as U0–4/C0–3/V4 [3]. A study involving 67 patients with vaginal atresia found that 25 patients (37.3%) had associated lower vaginal atresia, 36 patients (53.7%) had complete vaginal atresia, 1 patient (1.5%) had upper vaginal atresia, and 5 patients (7.5%) had vaginal apex atresia [4]. To facilitate clinical diagnosis and communication, the Obstetrics and Gynecology Branch of the Chinese Medical Association further classifies vaginal atresia into distal vaginal atresia and complete vaginal atresia. The former features a normally developed upper vaginal segment, cervix, and uterine body, with functional endometrium, while the latter often involves cervical developmental abnormalities, with a normally developed uterine body or a malformed uterine body with a functional endometrium [5].

Globally, vaginal atresia is often not diagnosed until puberty, with patients typically presenting due to primary amenorrhea or cyclical lower abdominal pain. Reports indicate that in the United States, vaginal atresia is the second most common cause of primary amenorrhea in tertiary care centers [6,7]. Furthermore, due to the complexity of its diagnosis, the actual incidence may be underestimated. Most cases of vaginal atresia are identified during puberty due to the absence of menstruation or difficulties with sexual intercourse, particularly in developing countries, where a lack of relevant knowledge often leads to delayed diagnosis. In China, the median age at diagnosis of vaginal atresia is approximately 12 years [8].

## 2. Etiology: Developmental Processes, Genetic Factors, and Environmental Influences

The embryonic origin of the vagina remains unclear, with four main hypotheses proposed. The most widely accepted theory suggests the upper two-thirds of the vagina develops from the Müllerian ducts, while the lower one-third arises from the urogenital sinus [9]. This hypothesis suggests that the upper two-thirds of the vagina develop from the Müllerian ducts, while the lower one-third arises from the growth and canalization of the urogenital sinus epithelium. The second hypothesis posits that the vagina originates entirely from the Müllerian ducts, with both the vaginal bulb and vaginal plate derived from this structure [10,11]. The third hypothesis combines the Müllerian and Wolffian duct origins, suggesting that the vagina develops from both the Müllerian and Wolffian ducts [12,13]. Finally, the “urogenital sinus origin” model asserts that the squamous epithelium of the cervix and vagina arises from the urogenital sinus, proposing that the squamous epithelium grows upward, replacing the columnar epithelium of the Müllerian ducts. All of these theories are based on anatomical and histological observations, with no definitive evidence confirming the embryonic origin of the vaginal epithelium. Recent lineage-tracing studies in mice support this “Müllerian duct + urogenital sinus” hypothesis, showing that the vaginal epithelium entirely originates from the Müllerian duct, with no contribution from the Wolffian duct [14].

Additionally, disorders of sexual development, such as androgen insensitivity syndrome (AIS) in XY individuals and congenital adrenal hyperplasia (CAH) in XX individuals, provide valuable insights into the processes of vaginal development. In individuals with complete AIS, despite having an XY chromosomal pattern, female external genitalia and a blind-ending vagina may develop, but the proximal vagina, cervix, and uterus are absent [15]. In contrast, XX females with CAH, who are exposed to excess androgens, retain a female-structured upper vagina and uterus, but exhibit abnormal external genitalia, ranging from a mildly enlarged clitoris, reduced vaginal opening with a separate urethral opening, and posterior labial fusion, to complete male virilization with a phallus, complete fusion of the labial folds, a single urethral opening at the glans penis, and a normally formed but empty scrotum [16]. These conditions help to further elucidate the origin of the vagina, suggesting that its development involves contributions from both the Müllerian ducts and the urogenital sinus, thereby advancing our understanding of the embryonic origin of the vagina.

Due to the lack of large pedigrees suitable for linkage analysis, researchers have employed association studies, functional studies, gene expression analysis, animal experiments, and bioinformatics research to screen for relevant genes and assess their pathogenicity in vaginal atresia.

Gene mutations in genes such as *TBX6* and the *Tyro3 RTK* family (including *Tyro3*, *Axl*, and *Mer*) are linked to distal vaginal atresia. The TBX family of transcription factors plays important roles in development, and *TBX6* has been identified as a potential gene involved in vaginal development [17], though further research is needed. Similarly, in animal models, mutations in *TBX3* and the *Tyro3 RTK* family have been associated with vaginal atresia [18,19]. In particular, *Mer* appears to be the most crucial gene in this family for vaginal development, with *Axl* and *Tyro3* possibly supporting its function [20]. In addition, a spontaneous point mutation in the *Lhfpl2* gene in mice has been shown to cause abnormal vaginal septum formation, the underdevelopment of the lower vagina, and normal ovary and uterus development [21]. As well as this, *Adamts18* plays a role in regulating the vaginal opening by affecting the fusion of the Müllerian ducts and the apoptosis of vaginal cells in mice [22].

Research has also identified chromosomal abnormalities associated with vaginal atresia. For example, a study found a uniparental disomy on chromosome 2 in some cases of distal vaginal atresia [8], which may affect genes involved in apoptosis, immune response, and tissue development. Additionally, duplications on chromosome 17q12, including the genes *HNF1B* and *LHX1*, have been linked to vaginal atresia. *HNF1B* is particularly important for urogenital development [23,24,25]. Insertions on chromosome 21q22.3q23.3 might also contribute [26]. These findings suggest that chromosomal abnormalities may play a role in the development of vaginal atresia, alongside other genetic mutations.

Despite the current progress in understanding the genetic factors associated with vaginal atresia, and the establishment of animal models to study the condition, the genetic background of vaginal atresia remains poorly understood. Further research is needed to continue elucidating the complex genetic mechanisms underlying this congenital anomaly. Currently, the field of genetic diagnosis for congenital malformations is developing rapidly. Early diagnosis of congenital defects can significantly improve diagnostic accuracy and help tailor personalized treatment plans, ultimately improving neonatal outcomes.

Environmental factors may also contribute to vaginal atresia by influencing gene expression or physiological events during development [27]. Maternal health status, nutritional levels, medication use, or exposure to specific environmental toxins during pregnancy could all impact the development of the fetal reproductive system. For instance, prenatal exposure to steroid medications may interfere with gonadal development, thereby increasing the risk of vaginal atresia. Due to maternal estrogen stimulation of the uterus and cervical glands, distal vaginal atresia may manifest during the neonatal period [28]. However, the mechanisms and specific associations of these environmental factors require further investigation for clarification.

## 3. Clinical Manifestations

Patients with vaginal atresia have a 46,XX karyotype, with partial or complete underdevelopment of the vagina. The uterus and cervix may be normal or exhibit fusion or resorption defects, with the distal or entire vagina replaced by fibrous tissue. At puberty, these patients may present with primary amenorrhea, cyclic pain, and pelvic masses. If retrograde menstruation is not detected in time, it can lead to endometriosis and pelvic adhesions [29,30,31,32].

In cases of congenital distal vaginal atresia, due to normal endometrial development and functional endometrium, menstrual blood cannot exit at menarche, resulting in early hematocolpos formation. This manifests as a large hematoma in the upper vagina, which can lead to hematocervix, hematometra, and hemoperitoneum in severe cases. On rectal examination, the mass is typically located low. Symptoms present early with severe abdominal pain and prominent masses, leading patients to seek medical attention early, making surgery less complicated and prognosis favorable [8]. However, some researchers note that a portion of distal vaginal atresia patients may exhibit severe manifestations in the neonatal period, including obstructive uropathy, intestinal obstruction, urinary tract infections, or systemic sepsis [33].

Congenital total vaginal atresia is often associated with cervical malformations, though uterine development is generally preserved and functional endometrium is present. Consequently, symptoms appear later, with milder abdominal pain and less obvious masses, often leading to delayed medical consultation [34]. Complete vaginal atresia may result in hematosalpinx, hemoperitoneum, and pelvic endometriosis. In a cohort study by Karina et al., 66.7% of patients with distal vaginal underdevelopment had endometriosis, and 100% of patients with cervical and vaginal underdevelopment had endometriosis [31].

Vaginal atresia is frequently associated with other malformations. These may include other congenital Müllerian duct anomalies, such as a unicornuate uterus and septate uterus, urogenital anomalies like renal dysplasia and congenital vesicovaginal fistula, and other congenital malformations such as scoliosis, cardiac anomalies, polydactyly, and anal atresia.

## 4. Diagnosis and Differential Diagnosis of Vaginal Atresia

The diagnosis of vaginal atresia involves a comprehensive approach, incorporating clinical manifestations, gynecological examination, imaging, and laparoscopic exploration to confirm the atresia location, type, and complications. The diagnostic process is crucial, as early identification and appropriate treatment options directly impact the patient’s quality of life and fertility potential. Clinical manifestations and gynecological examination are often the initial diagnostic clues, primarily including primary amenorrhea, cyclic abdominal pain, pelvic masses, and the absence or closure of the vaginal introitus. However, detailed diagnosis often requires imaging assistance, such as pelvic ultrasound or magnetic resonance imaging (MRI) [2]. Pelvic ultrasound is relatively accessible and easier to perform, though it has higher rates of missed or misdiagnosis. MRI’s superior soft-tissue contrast and multiplanar capabilities make it the gold standard for evaluating complex uterine malformations [35]. Finally, due to the limitations of clinical manifestations, gynecological examination, and imaging, laparoscopy may be performed when necessary to determine conditions such as endometriosis.

Conditions commonly confused with vaginal atresia include imperforate hymen and Mayer–Rokitansky–Küster–Hauser (MRKH) syndrome. Imperforate hymen primarily results from uncanalized urogenital sinus tissue, with less abdominal pain than in vaginal atresia patients, and the mass typically located in the vagina rather than the pelvis. Due to hematocolpos, patients with imperforate hymen may exhibit a bluish-purple hymen [36]. MRKH syndrome patients typically present with an absent uterus and vagina, though some with functional endometrium may exhibit primary amenorrhea and cyclic lower abdominal pain. Gynecological examination reveals only a shallow vaginal dimple without hematocolpos [37]. Though MRI, ultrasound, and other imaging techniques may confuse vaginal atresia with these two types of genital anomalies, clinicians should carefully assess clinical manifestations and gynecological findings to achieve a more precise differential diagnosis. However, their treatment differs: vaginal atresia primarily requires surgery, while MRKH syndrome typically involves vaginal dilation first. Differences in uterine development mean vaginal atresia patients more frequently need obstruction relief. Misdiagnoses and unclear distinctions in the literature, particularly between distal and complete vaginal atresia, can lead to treatment delays and inaccuracies. Scholars are urged to clearly classify and describe these conditions to improve diagnosis, treatment, and academic communication, ultimately advancing research and clinical practice. Comparisons of vaginal atresia, MRKH Syndrome, and imperforate hymen are summarized in Table 1.

Finally, diagnostic delays in developing countries should also be noted. In these regions, limited access to healthcare, a lack of awareness among healthcare providers, and cultural stigmas surrounding reproductive health contribute to delayed diagnosis and treatment. The shortage of trained medical professionals and inadequate diagnostic tools often prevent early detection. To address these barriers, just like other disease requiring early diagnosis, reducing stigmas, increasing public awareness improving training for healthcare providers, and expanding access to diagnostic services in rural and underserved areas are crucial steps [38]. Increasing financial access in these regions might also be crucial [39].

## 5. Treatment of Vaginal Atresia

The first-line treatment for patients with vaginal atresia is surgical intervention to relieve the obstruction [40]. For all types of vaginal atresia, the primary goals of surgery are to alleviate the obstruction, restore anatomical function, and prevent recurrence. However, given the varying degrees of uterine and cervical development in patients with different levels of vaginal atresia, as well as differing fertility requirements, specific treatment strategies—such as the choice of surgical approach and the material used to cover the newly created vaginal surface—may differ and often require individualized consideration.

### 5.1. Surgical Options for Distal Vaginal Atresia

Surgery for distal vaginal atresia has a high success rate and generally involves opening the atretic segment and draining menstrual blood from the upper vagina. The treatment for distal vaginal atresia primarily involves surgical incision of the atretic vaginal segment and relieving the obstruction. Surgery is not strictly required during menstruation, but if there is hematocolpos, the surgery should be performed as soon as possible to relieve the obstruction. First, perform a puncture to drain the blood and clarify the direction before incising the atretic part, and then try to dilate the incision. The reconstructed vagina should be able to accommodate two fingers. If the atretic segment is short and the wound is small, the vestibular mucosa can be sutured to the vaginal upper segment mucosa to create a continuous vaginal canal. For larger wounds, after hemostasis, a mold, amniotic membrane, or artificial biological graft can be used as a stent, and the area will be left to epithelialize postoperatively.

A modified “balloon vaginoplasty” provides a novel treatment option for patients with distal vaginal atresia. Initially developed by Saman et al. in 2009, balloon vaginoplasty differs from the traditional “cut-through” approach that involves dissection of the vesicorectal space [41,42]. The key feature of this technique is the use of a soft, non-erosive Foley balloon for traction, allowing the creation of a new vaginal canal by expanding the natural vaginal mucosa without dissecting the vesicorectal space [43,44,45]. Although Saman et al. demonstrated the adaptability of balloon vaginoplasty for various sacroiliac abnormalities, they did not specifically apply this method to patients diagnosed with vaginal atresia, nor did they clarify if this technique could effectively expand the distal vagina or thin the septum to reduce the risk of stenosis.

Building on this foundation, Zhang et al. modified balloon vaginoplasty in a 2022 study, demonstrating its ability to further expand the distal vagina and thin the septum in patients with vaginal atresia, reducing the risk of stenosis [46]. This modification provides a new option for patients with distal vaginal atresia, particularly those with obstructions ≥3 cm in length. Specifically, the method recommends a two-stage surgical approach. In the first stage, the surgeon inserts a Foley catheter through the obstructed vaginal canal until it reaches the hematocolpos in the upper vagina, inflating the balloon with 30–50 mL of saline to provide traction. The patient, with parental assistance, continues traction at home, pulling down the catheter by at least 1 cm each time, 6–8 times daily for 5–10 min per session, until only 1 cm of the obstruction remains.

The second stage involves the surgeon pulling down the balloon and making a “Z”-shaped incision to open the atretic vaginal segment, followed by postoperative stent placement to prevent retraction. Zhang et al. reported that the modified vaginoplasty was successful in patients with distal vaginal atresia, with no intraoperative complications and satisfactory postoperative outcomes.

### 5.2. Surgical Approaches for Complete Vaginal Atresia

Complete vaginal atresia is often associated with cervical dysgenesis, and the primary consideration in treatment is the possibility of preserving the uterus. If uterine preservation is feasible and the patient and family strongly desire to retain fertility, they should be fully informed about the extremely low postoperative pregnancy rate [47]. The surgery to preserve reproductive function involves creating a cervical-like canal between the uterus and vagina to allow unobstructed menstrual flow. With advances in surgical techniques and new materials, an increasing number of successful cases of fertility-preserving surgeries have been reported. In general, these procedures are divided into cervicovaginal canalization and uterovaginal anastomosis.

Cervicovaginal canalization involves creating a channel through the dense fibrous tissue between the uterine cavity and vagina. After the surgery, a cervical stent and vaginal mold are typically placed to prevent re-adhesion of the newly formed cervix and vagina [48]. Uterovaginal anastomosis is primarily used in cases with complete cervical atresia or when cervical tissue is insufficient for formation. This procedure entails the thorough dissection of the uterorectal and vesicouterine spaces, removal of dysplastic cervical tissue, and downward mobilization of the uterus, which is then anastomosed to the newly formed vaginal or vestibular mucosa [49]. A cervical catheter is often placed within the anastomotic site to ensure drainage and prevent adhesions.

Thirty-three studies have reported outcomes of cervicovaginal anastomosis in 53 patients with cervical and vaginal atresia. Of these, 33 patients (62.3%) resumed menstruation postoperatively, with one patient achieving natural pregnancy. Twelve patients experienced re-obstruction, and four died due to septic shock. Additionally, 21 studies reported outcomes of uterovaginal anastomosis in 121 patients, 92 of whom had cervical atresia with partial or complete vaginal atresia. Postoperatively, 94 patients (77.7%) resumed menstruation, with 6 achieving natural pregnancy. Eight patients experienced various complications; one developed a ureterovaginal fistula, and seven eventually underwent hysterectomy due to complications.

Lastly, studies indicate that postoperative vaginal stenosis is common following surgical correction of vaginal atresia, especially in patients with longer obstruction distances [48]. Therefore, regardless of the technique and materials used for reconstruction, the use of a vaginal dilator or mold for at least six months postoperatively is crucial to prevent vaginal stenosis and re-obstruction [50,51]. Patients who regularly used vaginal dilators postoperatively achieved significantly longer vaginal lengths and more satisfactory sexual outcomes compared to those with irregular usage [52].

### 5.3. Postoperative Care

The frequency of vaginal dilation or use of vaginal molds post-surgery depends on the length of the atretic vagina, in order to prevent re-closure or stenosis. A vaginal mold should be used regularly until the vaginal wound is fully epithelialized, which may take 3 to 6 months or longer, after which it can be used intermittently or replaced by self-dilation until regular sexual activity is resumed.

## 6. Applications and Advances of Different Materials in Upper Vaginal Atresia

For patients with high-level vaginal atresia, a lining for the newly constructed vagina is often required to promote epithelialization. Various materials have been used as vaginal coverings in vaginoplasty, including skin flaps, amniotic membrane, peritoneum, intestinal segments, oral mucosa, and biological grafts [53]. However, skin flaps are less commonly used today due to esthetic concerns, and oral mucosa is rarely applied in vaginal atresia cases. Currently, commonly used materials include an amniotic membrane, peritoneum, intestinal segments, and biological grafts. Advances and challenges of different materials in vaginal atresia are summarized in Table 2.

### 6.1. Biological Graft Vaginoplasty

In tissue engineering, extracellular matrix (ECM) materials are being explored for vaginal reconstruction. These materials provide an ECM-like environment for vaginal cell adhesion, ECM secretion, and host cell remodeling [54]. Although ECM-based grafts show promise, they remain largely experimental [55]. The only specific biological graft application reported for vaginal atresia patients involves the work of Papastefan et al., who utilized a small intestinal submucosa (SIS) ECM for vaginoplasty [56]. Their study indicated that SIS is an effective and safe alternative for vaginal reconstruction, with low perioperative morbidity and no need for long-term mold usage, while maintaining functional vaginal length. Following the creation of a neovaginal space, the SIS ECM was wrapped in a silicone mold, which was removed on the seventh postoperative day. The median surgery duration was 171 min, with an estimated blood loss of 10 mL and a 2-day hospital stay. At the last follow-up, the average vaginal length was 8.97 cm, with all patients successfully progressing to self-dilation or intercourse to maintain patency. However, the study lacked detailed outcomes on sexual satisfaction and comparison with alternative techniques.

### 6.2. Peritoneal Vaginoplasty

In peritoneal vaginoplasty, the peritoneum, along with portions of the bladder and rectal serosa, is used to cover the newly constructed vaginal surface. This approach is cost-effective and efficient, with high patient satisfaction regarding sexual quality, which can be further enhanced with postoperative interventions [57]. Peritoneal vaginoplasty can be performed via open surgery, vaginal approach, or laparoscopic approach. A recent prospective study reported outcomes of laparoscopic peritoneal vaginoplasty in nine patients with congenital cervical and complete vaginal atresia, using peritoneal flaps and cervical reconstruction. After a median follow-up of 48 months, the median neovaginal length was 7.5 cm, with three cases of postoperative complications that were resolved with appropriate treatment. All married patients reported satisfaction with their sexual experience, further demonstrating the suitability of the peritoneal approach for complete vaginal atresia patients [58].

### 6.3. Intestinal Vaginoplasty

Intestinal vaginoplasty has several advantages, including self-lubrication, growth compatibility for prepubescent patients, and a minimal risk of stenosis [59]. Traditionally, intestinal vaginoplasty has been performed via open surgery, but recent innovations include robot-assisted ileal vaginoplasty. One report described a robot-assisted ileal vaginoplasty after failed oral mucosa vaginoplasty in a patient with vaginal atresia. The procedure, performed through a combined abdominoperineal approach, resulted in successful recovery, and cystoscopy at 2 weeks, 3 months, and 1 year postoperatively confirmed adequate vaginal depth and successful expansion [60]. Some authors also propose reconstructing the vagina with an ascending colon segment, using the appendix to reconstruct the cervix. In this approach, the appendix connects the neovagina and uterus, maintaining cervical glands that may resist infection. This setup allows menstrual flow through the neovagina and enables sperm entry to the uterus, though pregnancy outcomes were not reported [61].

### 6.4. Amniotic Membrane Vaginoplasty

Amniotic membrane vaginoplasty is an affordable, safe, and simple technique that reconstructs or repairs vaginal structures using the amnion (a component of fetal membranes) as a biological material [62]. This method has a high success rate, with low postoperative pain, infection, and scarring, making it especially beneficial in resource-limited countries. Avsar et al. reported successful outcomes using human amniotic membrane in patients with vaginal atresia [63]. However, long-term follow-up showed that the McIndoe technique combined with amniotic membrane grafting did not fully restore normal vaginal function and anatomy in cases of congenital vaginal atresia [64]. Only one case of amniotic membrane vaginoplasty for a 15-year-old girl with vaginal atresia and cervical dysgenesis has been reported, though long-term follow-up data for this case is lacking [65].

## 7. Treatment for Patients with Co-Occurring Anomalies

For patients with coexisting anomalies such as rectovaginal fistula or anal atresia, the anatomical structures in proximity to vaginal atresia require careful consideration in surgical planning. Kisku et al., in a review of seven cases, emphasized the importance of appropriate timing and surgical approach when managing combined rectovaginal fistula and vaginal atresia. They suggested using the rectovaginal fistula as a new anus rather than a neovagina and advocated for delayed intestinal vaginoplasty to better assess uterine function [66]. Similarly, Pandya et al. explored the joint issue of vaginal and anal atresia, noting that while surgical repair generally yielded satisfactory outcomes, long-term bowel control often remained suboptimal [67]. Skerritt et al., through a literature review, evaluated the long-term reproductive and sexual function in patients with anorectal malformations and vaginal atresia, highlighting the need for more long-term data to support the superiority of specific vaginal replacement techniques [68].

## 8. Psychological Status

The bidirectional relationship between psychological issues and sexual dysfunction in patients with vaginal atresia has long attracted scholarly attention [69]. Specifically, sexual function scores are positively correlated with self-esteem scores and negatively correlated with depression and anxiety scores. As a condition impacting female sexual function, vaginal atresia also significantly affects the psychological state of patients. Studies have shown that among all patients diagnosed with complete vaginal atresia, 58.7% exhibit moderate to severe depressive symptoms. Factors associated with depressive symptoms in patients with complete vaginal atresia include age, parental attitudes, familial discord, peer bullying, sexual dysfunction, fertility anxiety, and discomfort associated with vaginal molds [70]. Although patients with distal vaginal atresia in China have been reported to attend mainstream schools and live independently [8], there is currently a lack of in-depth studies regarding their psychological status. During the treatment process, vaginal atresia patients require psychological preparation when using vaginal dilators postoperatively. Furthermore, patients with MRKH syndrome, which shares phenotypic similarities with vaginal atresia [71,72], are significantly affected by reproductive tract anomalies, exhibiting characteristics such as low self-esteem and depressive symptoms. Research indicates that preoperatively, 45.3% of patients report mild to moderate depression, while 34.0% report mild anxiety. Follow-up studies approximately three years postoperation show that while patients’ sexual arousal levels have significantly improved, their psychological health has not seen substantial improvement [72]. These findings underscore the importance of addressing the psychological state of vaginal atresia patients in both assessment and treatment, indicating that psychological intervention should play a critical role in their care. Psychological counseling can not only assist patients in coping with the psychological stress associated with their condition but also provide essential support before and after surgery, enhancing their confidence in treatment and offering avenues for adjusting expectations and accepting reality. Future research should further explore optimized approaches to psychological intervention, assess the long-term impacts of different methods on patients’ psychological states, and investigate how to better integrate these interventions into existing treatment plans for comprehensive physical and psychological rehabilitation.

## 9. Fertility Outcomes and Sexual Function

The fertility outcomes and sexual function of patients with vaginal atresia are important indicators for assessing treatment efficacy and quality of life. For patients with distal vaginal atresia, the relatively mild nature of the condition allows for surgical restoration of the vagina, which is usually sufficient to support sexual activity, with most patients achieving natural conception and experiencing unaffected pregnancy outcomes. In contrast, patients with complete vaginal atresia face potential complications due to cervical development anomalies, as well as surgical considerations regarding whether to excise the uterus and the specific method of vaginal formation, leading to less optimistic fertility outcomes [73]. Research by Reham et al. indicates that only 28.5% of patients with cervical-vaginal atresia achieved clinical pregnancy postoperatively, with a mere 14% resulting in live births [74]. Nonetheless, efforts to enhance fertility outcomes in patients with complete vaginal atresia continue. Recent studies indicate that vaginoplasty combined with cervical-vaginal anastomosis has a protective effect on the fertility of patients with complete vaginal atresia accompanied by cervical canal obstruction. For vaginal atresia patients with well-developed uteri and a desire for fertility, cervical-vaginal anastomosis should be prioritized [75]. Additionally, even patients who undergo vaginoplasty can improve their intrauterine environment prior to in vitro fertilization–embryo transfer (IVF-ET) through hysteroscopic evaluation and uterine dilation, ultimately achieving favorable pregnancy outcomes [76]. These findings suggest that advancements in surgical techniques and collaboration among gynecological, obstetric, reproductive, and neonatal departments may lead to continuous improvements in pregnancy outcomes for patients with cervical-vaginal atresia.

## 10. Conclusions and Future Directions

This paper reviews the epidemiology of vaginal atresia, exploring its causes from developmental, genetic, and environmental perspectives. It highlights the role of genetic susceptibility and environmental interactions in disease manifestation. The article summarizes clinical features, diagnostic methods, and treatment strategies for distal and complete vaginal atresia, as well as important case studies and recent clinical research. Psychological issues, fertility outcomes, and sexual function are also examined. While research has covered clinical symptoms and diagnoses well, there is a lack of focus on genetic factors, treatment options, and patient psychological states, which requires further investigation.

Genetic studies have identified several genes, including those from the *TBX* and *Tyro3 RTK* families, as contributors to vaginal atresia, though the exact mechanisms remain unclear. Larger cohort studies and animal models, such as gene knockout mice, are needed to explore other genetic factors and their pathways. Additionally, technologies like whole-exome and whole-genome sequencing offer valuable tools for exploring complex genetic backgrounds, which could enable the identification of genetic variants associated with vaginal atresia and facilitate the discovery of new pathogenic genes to better elucidate the disease’s pathogenesis, and furthermore inform genetic testing and targeted therapies in the future.

Regarding treatment, various surgical options exist, but large-scale studies and randomized controlled trials (RCTs) are lacking to determine the most effective and long-lasting interventions. Future research should focus on optimizing treatment protocols and exploring new biomaterials for vaginal reconstruction, with the goal of improving patient outcomes.

The psychological impact on patients with vaginal atresia, including coping with their condition and social pressures, is another important area that requires more attention. Incorporating psychological support into treatment plans is crucial. Furthermore, while pregnancy outcomes for these patients are often poor, especially for those with complete vaginal atresia, long-term follow-up studies are needed to explore influencing factors and improve reproductive health.

In conclusion, research on vaginal atresia should address genetic factors, treatment strategies, and the impact on patients’ psychological well-being and fertility outcomes. By advancing understanding in these areas, we can improve treatment and enhance patient quality of life.

## Figures and Tables

**Table 1:** Comparison of vaginal atresia, Mayer-Rokitansky-Küster-Hauser Syndrome (MRKH Syndrome), and imperforate hymen.

	Distal Vaginal Atresia	Complete Vaginal Atresia	MRKH Syndrome	Imperforate Hymen
Cause	Underdevelopment of the lower vagina, leading to an absent vaginal opening but a normal external genital appearance.	Complete vaginal closure, often associated with underdeveloped cervix and uterus.	Congenital absence of the vagina and uterus, often due to underdevelopment of the paired Müllerian ducts.	Failure of the hymen to perforate, leading to obstruction at the vaginal opening.
Clinical Manifestations	Normal external genitalia, no vaginal opening. Upper vagina may be dilated with blood accumulation.	Similar to lower vaginal atresia but often associated with cervical and uterine malformations.	Primary amenorrhea, normal external genitalia, shallow vaginal dimple (or no vaginal opening), absent or underdeveloped uterus.	Primary amenorrhea due to menstrual blood accumulation, with cyclic abdominal pain.
Vaginal Findings	No vaginal opening; mucosal surface of the upper vaginal segment appears normal.	Complete absence of the vaginal opening; may have vaginal bulging from blood accumulation.	Shallow vaginal dimple with no true vaginal canal.	Imperforate hymen with no vaginal opening, bulging hymen due to blood accumulation.
Abdominal Pain	Often severe due to hematocolpos (blood accumulation), leading to pelvic pain.	Severe abdominal pain from blood reflux and pelvic masses, including hematocolpos and possible endometriosis.	No menstrual blood obstruction; cyclical abdominal pain may occur only if a functional endometrium is present.	Mild abdominal pain due to blood retention in the uterus and/or fallopian tubes, usually less severe than in vaginal atresia.
Pelvic Mass	Palpable mass located anterior to the rectum, below the hymenal level.	Larger pelvic masses in the form of blood accumulation, affecting the uterus, fallopian tubes, and ovaries.	No significant pelvic mass, though a small palpable mass may be felt if a rudimentary uterus exists.	Palpable cystic mass in the vagina due to accumulated blood, possibly with protrusion of the hymen.

**Table 2:** Advances and challenges of different materials in upper vaginal atresia.

Vaginoplasty Technique	Advantages	Challenges/Considerations
Biological Graft Vaginoplasty	Promising in tissue engineering, provides ECM-like environment for cell adhesion and remodeling.	Still experimental, lack of long-term sexual satisfaction data.
Peritoneal Vaginoplasty	Cost-effective, high patient satisfaction, efficient. Can be carried out via open surgery, vaginal, or laparoscopic approach.	Postoperative complications in some cases, need for postoperative interventions to enhance sexual quality.
Intestinal Vaginoplasty	Self-lubricating, growth-compatible for prepubescent patients, low risk of stenosis.	Invasive surgery, complications in some cases, pregnancy outcomes not reported.
Amniotic Membrane Vaginoplasty	Affordable, safe, simple, minimal scarring and infection, suitable for resource-limited settings.	Long-term functional restoration not fully achieved, particularly for congenital vaginal atresia.
